# Pervaporation, Vapour Permeation and Membrane Distillation: From Membrane Fabrication to Application

**DOI:** 10.3390/membranes11030162

**Published:** 2021-02-26

**Authors:** Francesco Galiano, Roberto Castro-Muñoz, Alberto Figoli

**Affiliations:** 1 Institute on Membrane Technology, ITM-CNR, Via P. Bucci 17/c, 87036 Arcavacata di Rende (CS), Italy; 2 Tecnologico de Monterrey, Campus Toluca, Avenida Eduardo Monroy Cárdenas 2000, San Antonio Buenavista, Toluca de Lerdo 50110, Mexico

In recent decades, membrane technologies have attracted a lot of interest in operations for highly selective separations. Particularly, pervaporation (PV), vapour permeation (VP) and membrane distillation (MD) represent three membrane processes well-studied and applied at the research level and with a great potential of exploitation in different industrial sectors. These processes share the fact that the species to be removed are in the vapour phase during their permeation through the membrane, and the driving force is represented by a chemical gradient (pressure, temperature). Their field of application is wide and ever-expanding including biotechnology, pharmaceutical, food, chemical and petrochemical sectors, environmental protection and water treatment/desalination. This Special Issue entitled “Pervaporation, Vapour Permeation and Membrane Distillation: From Membrane Fabrication to Application” contains featured research papers dealing with the synthesis of membranes and their application in such processes.

The papers compiled in this Special Issue can be read as a response to the current needs and challenges in membrane development for azeotropic solvent separations, chemicals removal, water purification and desalination, mass transport, as well as process optimization with the help of experimental designs.

The papers included in this Special Issue are directed to fabricate high-performing membranes for PV, VP and MD processes, facing specific issues afflicting our society. Most of the contributors have described new concepts in membranes fabrication followed by their application. To date, the preparation of mixed matrix membranes (MMMs) is a current trend for the development of new membranes; several parameters play an important role in the separation performance of MMMs, such as filler types (e.g., geometry, shape, size, pore size, nature, etc.), polymer material, filler dispersion, among others. However, the understanding of mass transport in such MMMs becomes challenging since different materials are used. Today, there is a particular interest in theoretically estimating the mass transport and thus molecular permeation using modeling approaches, as documented by Zamani et al. [1]. Here, the authors described the effective permeability of MMMs with tubular fillers at steady-state concentration profile by means of Fick diffusion model.

To date, the research community has proved that the incorporation and coating of inorganic materials in polymer phases benefits the resulting membranes with better physicochemical properties impacting directly their separation performance. This was corroborated by Humoud et al. [2], who reported enhanced performance of carbon nanotube immobilized membrane (CNIM) for the treatment of high salinity produced water via direct contact membrane distillation. The CNIM loaded membranes showed improved water vapour flux and antifouling properties compared with the pristine ones, while the normalized flux decline with the polytetrafluoroethylene (PTFE) membrane after 7 h of operation was observed to be 18.2% more than the CNIM. In another contribution of this Special Issue, Amirah Idris and co-workers [3] also reported the improvement of intrinsic and mechanical properties of polysulfone (PSF) membranes by incorporating graphene oxide (GO). The membranes were fabricated via phase inversion method and successively tested in pressure retarded osmosis, demonstrating excellent water flux, salt reverse flux, high porosity and an enhanced microvoids’ morphology in comparison with the unfilled membranes. MD distillation is likely the second most explored technology after reverse osmosis for water desalination. One of the core application in this Special Issue comprises the study provided by Chiao et al. [4]. Their investigation concerns the overcoming of common issues in MD membranes, such as fouling and pore wetting. They developed a “graft to” electrospun zwitterionic bilayer membrane for the separation of hydraulic fracturing-produced water. Subsequently, the composite membrane was tested with an aqueous NaCl solution containing sodium dodecyl sulfate (SDS), an ampholyte and crude oil. With the presence of SDS and crude oil, the membrane showed a stable performance and salt rejection as high as 99.9%.

Safi et al. [5] also performed the water desalination with a porous poly (vinylidene fluoride-co-hexafluoropropylene) (PVDF-co-HFP) membrane using sweeping gas membrane distillation (SGMD), applying the Taguchi method for the optimization. The implementation of the design of experiment techniques was helpful for establishing the optimal separation conditions in terms of sweeping gas flow rate, temperature, feed concentration, and feed flow rate for getting the highest yield (in terms of permeation and salt rejection).

In an unprecedented application of MD, Alkhudhiri et al. [6] utilized the membrane technique for the removal of boron from water systems. In fact, air gap membrane distillation (AGMD) was able to remove up to 99% of the element. However, vacuum membrane distillation (VMD) exhibited good permeate flux (ca. 5.8 kg/m^2^) in comparison with the other MD technologies.

The exploration of novel nanoparticles into polymer membranes has been a current way to foster enhanced separations. Ultimately, metal-organic frameworks, so-called MOFs, represent a new class of inorganic materials exhibiting good properties and compatibility with polymers since they present organic sites for better contact with polymers. Msahel et al. [7] reported the evaluation of iron-based MOFs in polylactic acid membranes for the azeotropic separation of methanol (MeOH)/methyl tert-butyl ether (MTBE) solutions. This organic/organic mixture is one of the most challenging separations due to the complexity to break its azeotropic point via conventional distillation. In this work, it was demonstrated how the addition of iron-based MOFs was able to promote the permeation of methanol in polylactic acid membranes thus hindering MTBE transport at given conditions resulting in the organic/organic mixture separation. Similarly, Wang et al. [8] applied PV for the challenging removal of MTBE from water. Herein, the researchers utilized chemical modification protocols for the synthesis of mesoporous silica membranes silylated by fluorinated and non-fluorinated alkylsilanes. The strategic enhancement of the hydrophobicity in silylated silica membranes permitted the selective permeation and efficiency towards MTBE.

Finally, the role of membrane technology in absorption heat pumps was the objective of the review of Ibarra-Bahena et al. [9]. The authors focused on the advancement and replacement of membranes in the place of absorption and compression heat pump components. By reviewing the literature, the authors concluded that membrane devices can provide an opportunity to develop more compact and energy-efficient absorption heat pump systems at least at lab scale.

Over the course of this Special Issue, the editors have noticed the great efforts of contributors in providing real cases of study and compelling analysis of the state of the art. The papers are not only oriented to the development of new membranes but also in exploring their potential in different fields. This Special Issue has also evidenced that the application of PV, VP and MD can span from chemical separations to a plenty of processes and technologies in various fields, paving the way to their increasingly industrial exploitation.
